# Efficacy of ganglion impar block on vulvodynia: Case series and results of mid- and long-term follow-up

**DOI:** 10.1097/MD.0000000000026799

**Published:** 2021-07-30

**Authors:** Dae Gy Hong, Seong-Min Hwang, Jun-Mo Park

**Affiliations:** a Department of Obstetrics and Gynecology, School of Medicine, Kyungpook National University, Daegu, South Korea; b Department of Anesthesiology and Pain Medicine, Kyungpook National University Hospital, Daegu, South Korea; c Department of Anesthesiology and Pain Medicine, School of Medicine, Kyungpook National University, Daegu, South Korea.

**Keywords:** ganglion impar block, sympathetic, vulvar pain, vulvodynia

## Abstract

**Rationale:**

Vulvodynia is a common chronic gynecological disease that affects approximately 16% of women, although it is rarely diagnosed. However, no known effective treatment exists. The etiology of vulvodynia is unknown and may be heterogeneous and multifactorial, so it is difficult—if not impossible—to improve this condition using 1 treatment method. Reports have shown that vulvodynia has an element of neuropathic pain. Although the role of the sympathetic nervous system in neuropathic pain is controversial, sympathetic nerve blocks have long been used to treat patients with chronic pain giving good results. A ganglion impar block (GIB), a sympathetic nerve block technique, may effectively manage pain and discomfort in patients with vulvodynia.

**Patient concerns:**

Four patients suffering from chronic vulvar pain for 6 months–10 years were referred by gynecologists. The gynecologists could not identify the cause of the chronic vulvar pain, and symptoms were not improving by conservative therapy with medication. Patients complained of various chronic vulvar pain or discomfort. The initial visual analog scale (VAS) scores were 8 or 9 out of 10, and Leeds assessment of neuropathic symptoms and signs pain scale score was more than 12 out of 24. The review of gynecological medical records confirmed whether they showed allodynia during the cotton swab test and hyperalgesia to pin-prick test.

**Diagnoses:**

All patients were diagnosed with vulvodynia.

**Interventions:**

All patients were treated with a GIB, once in 2 patients, 3 times in 1 patient, and 4 times (1 alcoholic neurolysis) in the other patient, under fluoroscopic guidance.

**Outcomes:**

After the procedures, the VAS score and the leeds assessment of neuropathic symptoms and signs (LANSS) pain scale score were decreased to less than 2 and 5, respectively, in all patients. Follow-up observations for 6 months–2 years revealed that 2 patients’ symptoms entirely or nearly entirely improved and did not require further treatment. The pain of the remaining patients were well controlled with medications only.

**Lessons:**

GIB is a good treatment option for patients suffering from chronic pain and discomfort caused by vulvodynia.

## 1 Introduction

In 2015, the International Society for the Study of Vulvovaginal Disease, International Society for the Study of Women's Sexual Health, and International Pelvic Pain Society updated the definition of vulvodynia as “vulvar pain of at least 3 months’ duration, without clear identifiable cause, which may have potential associated factors”.^[1]^ Vulvodynia has various forms of clinical symptoms, and potential factors associated with developing vulvodynia include comorbidities and other pain syndromes, genetics, hormonal factors, inflammation, musculoskeletal diseases, neurologic mechanisms, psychosocial factors, and structural defects.

In the United States, approximately 16% of respondents reported a history of chronic, burning, sharp knife-like pain, or pain on contact lasting at least 3 months. Nearly 7% was reported to be experiencing such problems at the time of the survey.^[2]^ This was proven in subsequent studies.^[3,4]^ Although the pain and discomfort caused by vulvodynia affect patients’ quality of life, their family, and intimate partner, it also becomes a significant burden on society and the healthcare system. Many women live with pain and sexual dysfunction without accurate diagnosis and proper management.^[5–8]^ This may be due to the diversity of vulvodynia, making its cause challenging to be determined. Also, if the cause of vulvodynia is determined, it is often difficult to treat. Unfortunately, a particularly effective method for treating vulvodynia is unknown.

Vulvodynia is widely accepted as neuropathic in origin.^[8,9]^ While the relationship between neuropathic pain and the sympathetic nervous system (SNS) is controversial, the relationship between the chronic pain and the SNS is well established.^[10,11]^ Sympathetic nerve blocks have been used to treat chronic pain long time ago.^[11–14]^ The ganglion impar (also known as Walther's ganglion) is the only unpaired ganglion of the SNS at the sacrococcygeal junction level.^[12]^ It conveys sympathetic efferent to and nociceptive afferent from the perineum, distal rectum, perianal region, the distal urethra and vulva/scrotum, and the distal third of the vagina and supplies sympathetic innervation of the pelvic viscera. There is a preliminary report that the ganglion impar block (GIB) effectively reduced the neuropathic component of chronic coccygodynia.^[15]^

Authors reports successful pain and discomfort management in 4 patients with vulvodynia by the GIB under fluoroscopy guidance.

## 2 Case report

Case 1 was a woman in her 60 second who complained of chronic pricking vulvar pain and an itching sensation lasting 2 years. She had hypertension and a history of 3 vaginal deliveries. Her chronic vulvar pain occurred suddenly without a specific cause, and the pain may extend to the inner upper thigh. She felt particularly uncomfortable sitting, making it challenging to sit for more than 10 minutes. The patient's gynecological medical records confirmed that she did not show allodynia during the cotton swab test but showed hyperalgesia to the pin-prick test.^[16,17]^ The initial visual analogue scale (VAS) score was 8 out of 10, and the Leeds assessment of neuropathic symptoms and signs (LANSS) pain scale score was 13 out of 24.^[18]^ We diagnosed her with generalized vulvodynia. She had been prescribed various medications, such as 300 mg gabapentin, 2.5 mg tibolone, promestriene cream, 40 mg oxycodone, 120 mg fexofenadine, and 0.5 mg estriol. However, it did not control the symptoms, and it was impossible to increase oxycodone's dose because of nausea. Ultrasound-guided bilateral pudendal nerve blocks were performed with a mixture of 5 mL of 0.5% lidocaine and 10 mg triamcinolone at each side twice every 2 weeks. The VAS score dropped from 8 to 4 and 3 after 2 and 4 weeks, respectively. Though the VAS score was reduced by half, she complained of considerable discomfort in daily life. The fluoroscopy-guided GIB with a mixture of 5 mL of 0.5% lidocaine and 20 mg triamcinolone was performed. At the outpatient visit 2 weeks after the procedure, the VAS and the LANSS pain scale score dropped to 0, and the patient stopped complaining. No adjuvant treatment was performed, and the condition remained the same when she revisited after 6 months.

Case 2 was a woman in her 70 second who complained of chronic burning and stabbing vulvar pain and dysesthesia lasting 6 months. She reported that she started visiting a gynecologist 6 months ago because her symptoms were getting worse, but perhaps symptoms were minor, but they seemed to have existed before that time. She also reported having an gynecological operation a long time ago. She had hypertension, diabetes, depression, and Parkinsonism. She was once diagnosed and treated for somatoform disorder NOS. Her chronic vulvar pain occurred suddenly without a specific cause, and the pain may extend to the perineum. The gynecological medical records confirmed that she did not show allodynia during the cotton swab test but showed hyperalgesia to the pin-prick test. The initial VAS score was 8, and the LANSS pain scale score was 13. We diagnosed her with generalized vulvodynia. She had been prescribed various medications, such as 300 mg gabapentin, 2.5 mg tibolone, promestriene cream, 20 mg oxycodone, and 0.5 mg estriol, but it was unhelpful. A fluoroscopy-guided GIB with a mixture of 5 ml of 0.5% lidocaine and 10 mg triamcinolone to prevent excessive blood sugar rise was performed, and the VAS and LANSS pain scale score was reduced to 4 and 9 after 2 weeks, respectively. She did not feel much discomfort in daily life, even with a VAS score of 4. After, only drug treatment with 30 mg duloxetine, 150 mg pregabalin, and 0.5 mg estriol was performed. The VAS score and the LANSS pain scale score became 2 and 5, respectively, and were maintained continuously for 6 months. She remained with the same status for a year later without any treatment.

Case 3 was a woman in her 70 second who complained of chronic burning and pricking vulvar pain on contact, itching sensation, and dysesthesia lasting more than 10 years. She had a history of cervical cancer surgery 10 years ago and was accompanied both lower extremity lymphedema. For 10 years, she had been treated in numerous hospitals, but there was no improvement. There was no problem before the surgery. She could not wear tight clothes since the onset of symptoms. On the day of her first referral, she was wearing men's square briefs. Her chronic vulvar pain occurred suddenly without a specific cause, and the pain may extend to the perineum. The gynecological medical records confirmed that she showed allodynia during the cotton swab test and showed hyperalgesia to the pin-prick test. The initial VAS score was 9, and the LANSS pain scale score was 19. We diagnosed her with generalized vulvodynia. She had been prescribed various medications, such as 150 mg pregabalin, 2.5 mg tibolone, promestriene cream, 20 mg oxycodone, 120 mg fexofenadine, and 0.5 mg estriol, but it was unhelpful. The fluoroscopy-guided GIB with a mixture of 5 mL of 0.5% lidocaine and 20 mg triamcinolone was performed twice every 2 weeks. Her VAS score after each procedure reduced from 9 to 5 and 4 after 2 and 4 weeks, respectively. After the procedures, the VAS score was reduced by half, and the patient was delighted with the procedure's results. However, it was challenging to come to the hospital because the patient lived far from our hospital. It was as hard as the pain and discomfort caused by vulvodynia for her elderly husband to come to our hospital by public transportation over 3 hours with his wife. She has limited mobility due to lymphedema of both lower extremities. The patient wished for her husband and herself to maintain her current condition without frequent visits to our hospital. Chemical neurolysis should only be performed in limited quantities in patients suffering from cancer pain. However, the authors could not ignore the patient's situation. After listening to a sufficient explanation about the risk of chemical neurolysis and taking enough time to think about it for 2 weeks, the couple voluntarily decided to conduct chemical neurolysis. The same procedure was performed once again with 3 mL of 2.0% lidocaine, and alcohol neurolysis with 2 mL of 99% alcohol was performed for the fourth time. The concentration of lidocaine was increased to check whether motor nerves were included, and the amount of lidocaine was decreased to check for the effect of alcohol neurolysis, which will be performed afterward. After the procedures, the VAS scores were reduced from 4 to 3 and 2 after 2 and 4 weeks, respectively. Fortunately, the procedure was finished without any side effects, and the patient was pleased with the procedure's results. The patient also received fluoroscopy-guided bilateral lumbar sympathetic ganglion blocks twice to relieve both lower extremity lymphedema. After the procedures, the patient underwent drug treatment with 30 mg duloxetine, 150 mg pregabalin, and 0.5 mg estriol and has maintained the VAS score and the LANSS pain scale score of 2 and 5, respectively, for 2 years.

Case 4 was a woman in her late 30 second who complained of chronic burning, stabbing, throbbing, and electric shock-like vulvar pain on contact and dyspareunia 18 months ago. Symptoms have developed since receiving laparoscopic low anterior resection surgery and postoperative adjuvant radiation therapy 18 months ago. There was no problem before the surgery. After the surgery and postoperative adjuvant radiation therapy, she could not have a normal sex life with her husband due to severe dyspareunia during insertion. Also, she could not wear tight pants such as jeans. Her pain was confined to the vulvar vestibule area. The gynecological medical records confirmed that she showed allodynia during the cotton swab test and showed hyperalgesia to the pin-prick test. The initial VAS score was 9, and LANSS pain scale score was 19. We diagnosed her with localized, provoked vulvodynia (provoked vestibulodynia). She had been prescribed various medications, such as 60-mg duloxetine, 300-mg pregabalin, 16-mg hydromorphone, and 0.5-mg estriol from gynecologists, but it did not help to control the symptoms. The patient underwent fluoroscopy-guided GIB with a mixture of 5 ml of 0.5% lidocaine and 20 mg triamcinolone 3 times every 2 weeks. After the procedures, the VAS scores were reduced from 9 to 5, 4, and 2 after 2, 4, and 6 weeks, respectively. The VAS score and the LANSS pain scale score became 2 and 5, respectively, and were maintained continuously for 2 years. She underwent only drug treatment with 30 mg duloxetine, 150 mg pregabalin, and 0.5 mg estriol and had a normal sex life again.

**Figure 1:**
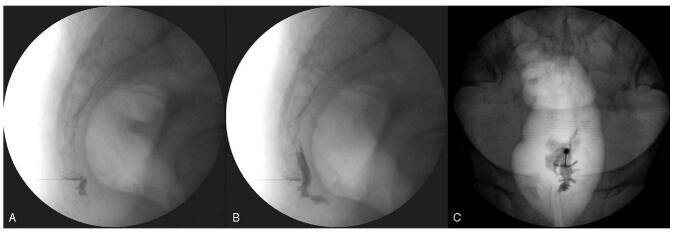
Ganglion impar block under fluoroscopic guidance. (A, B). The lateral fluoroscopy view shows “the reverse comma sign,” and the radiocontrast spread throughout the coccyx. (C). The anteroposterior fluoroscopy view shows that the radiocontrast covered both sides of the midline of the coccyx.

**Ganglion Impar Block:** All procedures were performed with informed consent under the Helsinki Declaration before implementation. All fluoroscopy-guided GIBs were performed using a trans-sacrococcygeal ligament approach technique.^[12]^ After placing the patient in a prone position on the C-arm table, the sacrococcygeal ligament area was identified under fluoroscopic guidance. The area was aseptically sterilized and draped sufficiently. The skin entry point of the 22G spinal needle was at the midline of the sacrococcygeal junction identified under fluoroscopic guidance. Infiltration was performed with a small amount of 2% lidocaine at the identified skin entry point. A spinal needle was inserted into the midline of the sacrococcygeal junction under fluoroscopic guidance. After penetrating the skin and dorsal sacrococcygeal ligament, passing through the sacrococcygeal disc, the needle tip was placed anterior to the ventral sacrococcygeal ligament using the loss of resistance technique. A radiocontrast (0.5 mL) was injected, and the correct position of the needle was confirmed using the “reverse comma sign” (Fig. 1A and B). Afterward, the radiocontrast was injected 1 or 2 more times to check if the drug can be accurately spread to the ganglion impar site using lateral and anteroposterior fluoroscopy views (Fig. 1B and C). A mixture of 0.5% lidocaine and triamcinolone was slowly injected after confirming all. No case of infection, anal incontinence, or unscheduled hospitalization was observed after the blocks.

Table 1 summarizes the clinical characteristics, assessment, treatment results and follow-up details of the 4 patients included in the case series. Approval of this study using retrospective chart review was waived from the Ethics Committee of Kyungpook National University Chilgok Hospital, based on their policy on case series. The authors obtained written consent from the patients to publish this case series.

**Table 1:** Summary of cases.

	Case 1	Case 2	Case 3	Case 4
Age	60 s	70 s	70 s	30 s
Chief complaint	pricking, itching	burning and stabbing, dysesthesia	burning and pricking on contact, itching, dysesthesia	burning, stabbing, throbbing, electric shock-like pain on contact, dyspareunia
Duration	2 yr	6 mo	more than 10 yr	18 mo
Past history	three vaginal delivery	one gynecological OP	cervical cancer OP 10 yr ago	Lap LAR and post-OP adjuvant RT 18 mo ago
Comorbidity	HTN	HTN, DM, depression and Parkinsonism	both lower extremity lymphedema	none
Initial VAS and LANSS score	8 / 13	8 / 13	9 / 19	9 / 19
Cotton swab test (allodynia)	–	–	+	+
Pin-prick test (hyperalgesia)	+	+	+	+
Type of vulvodynia	Generalized	Generalized	Generalized	Provoked vestibulodynia
GIB	1	1	4 (one neurolysis)	3
VAS and LANSS score after GIB	0 / 0	2 / 5	2 / 5	2 / 5
Present treatment	none	none	30 mg duloxetine, 150 mg pregabalin, 0.5 mg estriol	30 mg duloxetine, 150 mg pregabalin, 0.5 mg estriol
Follow up period	6 mo	18 mo	2 yr	2 yr

## 3 Discussion

There is no consensus on treatment algorithms for vulvodynia internationally, and recommended guidelines are primarily based on expert opinions, case series, and a limited number of placebo-controlled randomized clinical trials.^[19]^ The known most important methods for treating vulvodynia are oral therapies using antidepressants and anticonvulsants and topical therapies using ointment or cream.^[20]^ Also, injectable or nonpharmacologic therapies are tried; and surgical methods are sometimes tried as a last resort. Although all patients in this case series were prescribed various drugs by gynecologists, it did not help improve their symptoms. Although no definitive treatment for vulvodynia is known, a multidisciplinary approach is essential in treating vulvodynia.^[7,8,21]^

Women suffering from vulvodynia go to gynecologists and multiple healthcare providers in various fields. However, the clinicians they meet may not be familiar with vulvodynia, leading to incorrect diagnosis and treatment. In summary, normal vulvar appearance (with or without local erythema) and normal vaginal walls and secretions, in association with introital sensitivity to the cotton swab test, and pelvic floor muscle tenderness seen in some patients, can diagnose vulvodynia.^[19]^ However, in practice, accurately diagnosing vulvodynia is complex and requires considerable time and effort. Before diagnosing vulvodynia, it is essential to rule out other conditions that may cause similar pain. An accurate diagnosis must include a thorough medical history check with a particular focus on pain history, sexual history, and psychosocial assessment. Physical examinations, such as a careful visual examination of the vulva, a cotton swab test to determine painful sites, sensitive speculum examination to evaluate for secretions or abnormalities in the vaginal mucosa, and musculoskeletal examinations that focus on evaluating the pelvic floor muscles, must also be performed meticulously.^[22]^ For diagnosing patients included in this case series, details related to the nature and characteristics of pain and thorough review of past gynecological medical records were thoroughly checked at the pain clinic. However, the physical examination primarily referred to gynecological medical records. The VAS and LANSS pain scale scores were identified through the patient's direct expression under the direction of the pain physician at the pain clinic at each patient visit. It was only at the first and last meeting with gynecologists that patients were checked for allodynia during the cotton swab test and hyperalgesia to the pin-prick test. However, a thorough physical examination was not performed every time with gynecologists.

Although the incidence of vulvodynia is highest in younger women, vulvodynia can occur in women of all ages, including postmenopausal women.^[19]^ Women more than 70 years have a lower prevalence of vulvodynia than younger women, but sexually active women ≥70 years have a similar prevalence to younger women.^[3]^ Among the 4 patients included in this case series, 3 were elderly (in their 60 second and 70 second), and only 1 patient was in her late 30 second. The number of patients is small; therefore, accurate judgment cannot be made. However, perhaps this is because younger patients are more reluctant to accurately reveal the actual situation, even to gynecologists, even if they suffer from vulvodynia. Among the types of vulvodynia, provoked vestibulodynia is the most common type.^[3]^ However, it is seen that the generalized vulvodynia was more common in this case series because 3 of the 4 patients were elderly (in their 60 second or 70 second). Generalized unprovoked vulvodynia is less common and typically present in older women.^[23]^

Vulvodynia is characterized by mechanical allodynia and hyperalgesia localized in the vulvovaginal area.^[19]^ Mechanical hyperalgesia/allodynia and thermal hyperalgesia are common sensory signs in patients with different etiologies of neuropathic pain and surrogate animal and human models with neuropathic pain.^[24]^ Neuropathic mechanisms likely contributed to the patient's pain when the LANSS pain scale score is 12 or higher.^[18]^ Considering that the patients in this case series had a LANSS pain scale score of 12 or higher, it is thought that all patients’ pain in this case series should be treated as neuropathic pain. Neuropathic pain is caused by a lesion or disease of the somatosensory nervous system.^[25]^ According to this definition, vulvodynia cannot be designated as neuropathic pain. If vulvodynia is excluded from neuropathic pain, there is a risk that patients will be stigmatized for having a somatization disorder, which is neither a real nor obvious abnormality. However, they are suffering from a physical condition. Since this pain does not fit into the concept of neuropathic pain, it led to the use of other undefined descriptors of vulvodynia, such as dysfunctional or psychosomatic pain, which does not provide insight into possible mechanisms. It may also stigmatize patients causing only psychological suffering.

The relationship between neuropathic pain and SNS has not been fully elucidated. However, the relationship between chronic pain, such as vulvodynia and the SNS, is well established.^[10,11]^ Sympathetic nerve blocks have long been used to treat various chronic or neuropathic pain.^[11–14]^ Although the innervation of the ganglion impar is not accurately known, GIB was first described in 1990 by Plancarte et al to treat sympathetic pain of malignant origin, and it has been used to treat various diseases.^[12,26]^ Four techniques can be followed when performing GIB; they include the anococcygeal ligament, trans-sacrococcygeal approach, intercoccygeal joint approach, and paracoccygeal approach.^[12]^ All fluoroscopy-guided GIBs were performed using the trans-sacrococcygeal ligament approach technique. Regardless of which technique was used, knowing the exact location of the ganglion impar is essential to perform the procedure accurately. Oh et al reported that although the anatomical position of the ganglion impar is variable, it is located within 0.6 from the midpoint of sacrococcygeal joint, assuming that the distance from the midpoint of sacrococcygeal joint to the tip of the coccyx is 1.^[27]^ To confirm whether the drug to be injected will reach the ganglion impar, we injected the radiocontrast several times to check whether the radiocontrast reached enough from the sacrococcygeal joint to 0.6 before injecting the drug. In addition to GIB, a case report showed promising results by treating vulvodynia with lumbar sympathetic ganglion block, another kind of sympathetic block.^[28]^

All patients took medications only before the first visit. Each gynecologist prescribed slightly different medications, but they were unhelpful to the patients. In addition to the difficulty in treating vulvodynia, what is more problematic in this situation is the lack of communication between specialized subjects due to the specialization and subdivision of medicine. Most of the patients with vulvodynia see a gynecologist first. However, most gynecologists do not know much about other possible interventional treatment methods other than the known conservative treatment methods, even if they have an accurate diagnosis. Even if there are reports that GIB is effective, it is highly likely that many gynecologists are not aware of it. Vulvodynia remains a diagnosis of exclusion. Taking this into account, gynecologists play a significant role in excluding other causes of vulvar pain, screening for psychosexual and pelvic floor dysfunction, and co-working with other healthcare providers to manage pain in women.^[7]^ Therefore, a multidisciplinary approach is vital for treating patients with vulvodynia. The principle of treating patients with vulvodynia is that a multidisciplinary approach, including psychotherapy, pelvic physical therapy, and medical therapy, must be taken, and treatment tailored to individual characteristics must be performed.^[21]^

There are some limitations to the results of this case series. First, the number of patients included in this case series is only 4, so it is difficult to generalize the results. Second, since this study used a retrospective chart review, a thorough physical examination using the cotton swab test or tampon test was not performed every time a patient visited for follow-up. At every follow-up at the pain clinic, like other general patients visiting the pain clinic, the procedure results were confirmed using the VAS and LANSS score, which the patient directly expressed. However, in the case of pain physicians, it is also essential to consider that in most cases, the VAS score directly expressed by the patient is a commonly used method when determining the procedure's effectiveness. The tampon test's significance,^[29]^ known to be the best for confirming the degree of pain and effect before and after the procedure, respectively, was sufficiently explained. However, unfortunately, 3 patients in their 60 second or 70 second refused to take the tampon test because they did not have dyspareunia because they were not sexually active. The other younger patient in her late 30 second refused the tampon test for fear of experiencing the same severe pain. Despite these limitations of this case series, the authors believe that the results of this study will help physicians to consider GIB as one of several treatment options.

In conclusion, GIB can be an effective interventional treatment method for treating vulvodynia, and a multidisciplinary approach and multimodal treatment are essential to treat vulvodynia effectively.

## Author contributions

**Conceptualization:** Dae Gy Hong, Jun-Mo Park.

**Data curation:** Seong-Min Hwang.

**Formal analysis:** Dae Gy Hong, Jun-Mo Park.

**Investigation:** Seong-Min Hwang, Jun-Mo Park.

**Methodology:** Jun-Mo Park.

**Project administration:** Dae Gy Hong, Jun-Mo Park.

**Supervision:** Dae Gy Hong, Jun-Mo Park.

**Validation:** Dae Gy Hong.

**Writing – original draft:** Jun-Mo Park.

**Writing – review & editing:** Dae Gy Hong, Seong-Min Hwang, Jun-Mo Park.
